# Co-expression of S100A14 and S100A16 correlates with a poor prognosis in human breast cancer and promotes cancer cell invasion

**DOI:** 10.1186/s12885-015-1059-6

**Published:** 2015-02-13

**Authors:** Mizuko Tanaka, Naoki Ichikawa-Tomikawa, Namiko Shishito, Keisuke Nishiura, Tomiko Miura, Ayumi Hozumi, Hideki Chiba, Sayaka Yoshida, Tohru Ohtake, Takashi Sugino

**Affiliations:** 1 Department of Basic Pathology, Fukushima Medical University School of Medicine, Fukushima, 960-1295 Japan; 2 Department of Organ Regulatory Surgery, Fukushima Medical University School of Medicine, Fukushima, 960-1295 Japan; 3 Division of Diagnostic Pathology, Shizuoka Cancer Center Hospital, 1007 Shimonagakubo, Nagaizumi-cho, Sunto-Gun, Shizuoka, 411-8777 Japan

**Keywords:** S100A14, S100A16, Breast cancer, Prognosis, Invasion, Actin

## Abstract

**Background:**

S100 family proteins have recently been identified as biomarkers in various cancers. Of this protein family, S100A14 and S100A16 are also believed to play an important role in tumor progression. The aim of the present study was to clarify the clinical significance and functional role of these molecules in breast cancer.

**Methods:**

In a clinical study, an immunohistochemical analysis of S100A14 and S100A16 expression in archival specimens of primary tumors of 167 breast cancer patients was performed. The relationship of S100A14 and S100A16 expression to patient survival and clinicopathological variables was statistically analyzed. In an experimental study, the subcellular localization and function of these molecules was examined by using the human breast cancer cell lines MCF7 and SK-BR-3, both of which highly express S100A14 and S100A16 proteins. Cells transfected with expression vectors and siRNA for these genes were characterized using in vitro assays for cancer invasion and metastasis.

**Results:**

Immunohistochemical analysis of 167 breast cancer cases showed strong cell membrane staining of S100A14 (53% of cases) and S100A16 (31% of cases) with a significant number of cases with co-expression (p < 0.001). Higher expression levels of these proteins were significantly associated with a younger age (<60 years), ER-negative status, HER2-positive status and a poorer prognosis. Co-expression of the two proteins showed more aggressive features with poorer prognosis. In the human breast cancer cell lines MCF7 and SK-BR-3, both proteins were colocalized on the cell membrane mainly at cell-cell attachment sites. Immunoprecipitation and immunofluorescence analyses demonstrated that the 100A14 protein can bind to actin localized on the cell membrane in a calcium-independent manner. A Boyden chamber assay showed that S100A14 and S100A16 knockdown substantially suppressed the invasive activity of both cell lines. Cell motility was also inhibited by S100A14 knockdown in a modified dual color wound-healing assay.

**Conclusions:**

To our knowledge, this is the first report showing the correlation of expression of S100A14, S100A16, and co-expression of these proteins with poor prognosis of breast cancer patients. In addition, our findings indicate that S100A14 and S100A16 can promote invasive activity of breast cancer cells via an interaction with cytoskeletal dynamics. S100A14 and S100A16 might be prognostic biomarkers and potential therapeutic targets for breast cancer.

**Electronic supplementary material:**

The online version of this article (doi:10.1186/s12885-015-1059-6) contains supplementary material, which is available to authorized users.

## Background

Breast cancer is the leading cause of cancer mortality in women worldwide [1]. Significant advances in early detection and molecular-based treatments have improved breast cancer patient survival. Further understanding of the molecular mechanism of breast cancer progression would produce new biomarkers for the precise prediction of patient prognosis and for molecular targeted-therapy. We have focused on the detection of novel biomarkers of cancer progression including of invasion and metastasis based on experimental studies [2] and have identified the S100 family proteins S100A14 and S100A16 as important candidate molecules for the regulation of metastatic disease.

S100 proteins belong to a large subgroup of 25 small, acidic proteins that are characterized by distinctive homo- or hetero-dimeric architecture and EF hand Ca^2+^-binding motifs, and which are expressed in a variety of cell types. S100 proteins have a broad range of intracellular functions that are exerted through the modulation of their subcellular localization and interactions with specific target proteins responsible for cell growth, differentiation, motility, and cell-cycle regulation [3]. S100 proteins have recently become of great interest because of their close association with inflammation, neurodegenerative disorders and cancer [4,5].

S100A14, which was isolated by analysis of a human lung cancer cell line, is differentially expressed in a variety of cell types, in both normal and neoplastic tissues. S100A14 expression is up-regulated in cancers of the lung, breast, and uterus, whereas it is down-regulated in tumors of the colon, kidney, and rectum [6]. The overexpression of S100A14 was correlated with poorer prognosis in breast cancer and liver cancer [7-9], while it was correlated with favorable prognosis in colorectal and small intestinal cancers [10,11]. Several studies suggest that S100A14 affects cell proliferation, invasion and motility through interactions with HER2, MMP2, P53 and receptor for advanced glycation end products (RAGE) [9,12-15]. However, to date, the mechanism of its cell membrane localization and the precise role of S100A14 in human cancer remain unclear and controversial.

S100A16, a novel member of the S100 family that was isolated from an astrocytoma, has been proposed to be a binding partner of S100A14 [16,17]. S100A16 is ubiquitously expressed and is elevated in various tumors [18]. S100A16 is potentially relevant to malignancy. However, the physiological and pathological roles of S100A16, especially its roles in cancer, are largely unknown.

The first aim of the present study was to investigate the clinical significance of S100A14 and S100A16 expression in the prognosis of breast cancer patients. We analyzed the correlation of their protein expression with the prognosis of 167 breast cancer patients using an immunohistochemical method. The second aim was to explore the molecular mechanisms by which S100A14 and S100A16 contribute to the progression of breast cancer. We investigated the interactions of S100A14, S100A16 with other binding protein(s) in breast cancer cells and examined the function of these molecules in cellular proliferation, migration and invasion.

## Methods

### Patients and samples

The analysis of human tissues was approved by the Human Research Ethical Committee of Fukushima Medical University (No. 1203). Tissue samples from 167 patients who underwent surgical resection for primary invasive breast cancer at Fukushima Medical University Hospital from January 1990 to December 1996 were collected. After surgery, most patients were treated with the standard practice guidelines at that time and have been followed up regularly. Formalin-fixed, paraffin-embedded materials were used for routine staining with hematoxylin and eosin and for staining by immunohistochemical techniques.

### Immunohistochemical analysis

Rabbit polyclonal antibodies for S100A14 (Acris) and S100A16 (Proteintech) were used for this study. Specificity of the antibodies was determined by two methods. One method was an absorption test using S100A14 (Abcam) and S100A16 (Proteintech) recombinant proteins, in which the signal of each protein was diminished by the absorbed antibody in both immunohistochemistry and Western blotting (Additional file 1: Figure S1). Another method was an immunofluorescence study using MCF7, a human breast cancer cell line, which was transfected with the S100A14 or S100A16 expression vector described below, in which an increased signal for each protein on the cell membrane was detected using an immunofluorescence method with an antibody to each protein (data not shown). Immunostaining was performed by using an indirect streptavidin-biotin immunoperoxidase method (SAB-PO (M) kit, Nichirei Corp.). After antigen retrieval in a microwave oven for 15 min in 10 mM citrate buffer (pH 9.0 for S100A14 and pH 6.0 for S100A16), endogenous peroxidase activity was blocked with a 3% H_2_O_2_-methanol solution. The slides were incubated with primary antibodies (diluted 1/100) overnight at 4°C, washed with PBS, and then incubated with secondary biotin-labeled antibodies for 30 min at room temperature. Antibody localization was visualized with peroxidase-conjugated streptavidin for 30 min at room temperature, followed by the diaminobenzidine reaction. The slides were counterstained with hematoxylin.

### Cell lines and cell culture

The human breast cancer cell lines MCF7, ZR75-1, SK-BR-3 and MDA-MB-231 were purchased from the American Type Culture Collection. The cells were cultured in DMEM (MCF7 and SK-BR-3 cells) or RPMI1640 (ZR75-1 and MDA-MB231 cells) supplemented with 10% fetal bovine serum (FBS). Three-dimensional (3D) cultures of multicellular spheroids of MCF7 cells were grown in 8-well chamber slides (BD Biosciences) using a type I collagen gel (KOKEN) according to the manufacturer’s protocol (KOKEN). In order to determine whether the localization of S100A14 and S100A16 proteins is calcium-dependent or not, cells were incubated with 10 mM EGTA according to a previous method [19].

### Construction of S100A14 and S100A16 expression vectors and transfection

cDNA expression vectors for the human *S100A14 and S100A16* genes were constructed by PCR amplification of their coding regions using cDNAs derived from MCF7 cells as templates and specific primers, followed by cloning of the genes into a pEGFP expression vector (Takara-Clontech, Shiga, Japan). The primer sequences used for PCR were, *S100A14* forward: 5′-atgggacagtgtcggtcagccaacgca-3′, reverse: 5′-acccatgagctccccagagcatccaagac-3′ and S100A16 forward: 5′-agcagggagatgtcagactgctacacgga-3′, reverse: 5′-aggtgtggccaaaggggtctctagctg-3′. Specificity of these primers was determined by a homology search (Standard Nucleotide BLAST, NCBI). The constructed. plasmids containing *S100A14* and *S100A16* tagged with *GFP,* and the empty vectors of pEGFP-N1 and ptdTomato-N1 (Takara-Clontech), were introduced into MCF7 cells by using the FuGENE transfection reagent (Roche) according to the manufacturer’s protocol. To establish stable transfectants, selection of the cells was started 48 hours after transfection in 6-well plates with G418 antibiotics (0.8 mg/ml, Promega). Resistant cells were cloned by the single cell cloning method after 3 weeks of selection.

### RNA interference transfection

Stealth RNAi targeted to human S100A14 and S100A16, and RNAi negative control (Life Technologies) were used for RNAi experiments. Three sets of siRNAs with different sequences for each mRNA were purchased. For reverse transfection, 6 pmol RNAi duplexes were diluted in 0.1 ml Opti-MEM medium in each well of a 24-well plate. One μl Lipofectamine MAX reagent (Life Technologies) was added to the well. After 10 min incubation, 0.5 ml of MCF7 or SK-BR-3 cells (2 × 10^5^ cells /ml) were added to each well in DMEM with 10% FBS. The gene knockdown efficiency of RNAi was determined by immunofluorescence microscopy with anti S100A14 and S100A16 antibodies (Acris) (Additional file 2: Figure S2). The most effective siRNAs were used for the following experimental studies. The sequences of the siRNAs that were ultimately selected were: S100A14; 5′-GAGUUCAGGAGUUUCUGGGAGCUGA-3′ and S100A16; 5′-CCAAUCAUGAUGGGCGCAUCAGCUU-3′. The detection primers were: *S100A14* forward; 5′-atgggacagtgtcggtcagccaacgca-3′, reverse; 5′-aggcccacagtctctccccaacaccc-3′, S100A16 forward; 5′-cagggagatgtcagactgctacac-3′, reverse; 5′-catcaggccagtgcctggaa-3′. The specificity of these siRNAs and primers was determined by a homology search (Standard Nucleotide BLAST, NCBI).

### In vitro invasion assay

A cell invasion assay was performed in BioCoat cell culture inserts with a polystyrene membrane (8-μm pore; BD Bioscience) in a 24-well tissue culture plate. The culture insert was coated with Matrigel (BD Bioscience, 8.7 μg per chamber); the lower chamber was filled with DMEM containing 10% serum. A total of 4 × 10^5^ MCF7 cells or 1 × 10^5^ SK-BR-3 cells were seeded in the upper chamber containing DMEM with 10% serum and the cells were incubated at 37°C for 24 h. After wiping off the cells on the upper side, the membrane was removed and stained with Giemsa solution. The cells that had migrated to the lower side of the membrane were counted under a microscope.

### Wound healing assay and dual-color wound healing assay

To visualize the effect of transfected RNAi targeted against S100A14 and S100A16 on the motility of living cells, we performed two types of wound healing assays, the standard method and a modified dual-color wound healing assay. For the standard assay, a distinct area of the cell layer of a monolayer culture of MCF7 cells, which were transiently transfected with S100A14 and S100A16 siRNAs or with negative control siRNA, was wounded using a micropipette tip. The distance over which the cells had migrated into the wounded areas following 48 h incubation at 37°C was evaluated under a microscope. For the modified assay, we used MCF7 stable transfectants that were labelled with either green or red fluorescence and were additionally transfected with gene-specific siRNA or control siRNA, respectively. MCF7 cells that were stably transfected with either pEGFP (green) or ptdTomato (red) (Takara-Clontech) were first constructed as described above. Each transfectant was then further transfected with Stealth RNAi for human S100A14 or RNAi negative control, respectively. These green and red fluorescent cells were then mixed in equal numbers and plated at a density of 1×10^5^ cells per well in a 24-well tissue culture plate. After 48 h, a distinct area of the monolayer culture was wounded using a micropipette tip. Cells migrating into the scratched region over a period from 0 to 48 h were recorded by using a phase-contrast fluorescent microscope (Olympus).

### Immunoprecipitation

Protein extracts from MCF7 cells were prepared using HEPES buffer containing 1% NP-40. To determine whether the binding between S100A14 protein and actin is calcium-dependent or not, 0 or 2 mM CaCl2, or, 2 mM CaCl2 and 10 mM EDTA were added to the extracts. Rabbit anti-S100A14 antibody (diluted 1/100), anti-actin antibody (diluted 1/100) or normal rabbit IgG as the IP control (Sigma-Aldrich, St. Louis, USA) was applied to Protein A Sepharose beads (GE Healthcare) suspended in PBS-buffer and incubated for 2 h at room temperature. To cross-link antibodies to the beads, DMP (dimethyl pimelimidate dihydrochloride) in boracic acid was added and incubated for 30 min at room temperature. After washing with 0.1 M glycine-HCl (pH 2.8) to remove unbound antibody, the beads were incubated with protein extracts overnight at 4°C. The beads were washed with HEPES buffer, and protein was eluted with 0.1 M glycine-HCl (pH 2.0) for 1 h on ice.

### Immunofluorescence and western blot analyses

Cultured cells were plated on 8-well chamber slides for 24 to 48 h. Cells were fixed with 4% paraformaldehyde and permeabilized for 5 min at room temperature with 0.1% Triton X-100 in PBS, followed by blocking for 1 h with 5% skimmed milk. Cells were stained with rabbit polyclonal antibodies for S100A14 and S100A16 for overnight at 4°C, followed by incubation with FITC-labeled secondary antibody for 1 h at room temperature. To observe filamentous actin, cells were stained with rhodamine-conjugated phalloidin (Molecular Probes). The slides were mounted in mounting medium containing DAPI and analyzed using an inverted fluorescence microscope and by laser scanning confocal microscopy. Cellular proteins extracted with Cell lysis reagent (Sigma) were analyzed by Western blotting. Equal amounts of proteins were electrophoresed by standard SDS-PAGE under reducing conditions and were then transferred onto Immobilon membranes (Merck-Millipore). The target proteins were detected by immunoblotting using S100A14 and S100A16 antibodies (diluted 1/1,000) according to standard protocols using the ECL Advance Western Blotting Detection Kit (GE Healthcare).

### Statistical analysis

SPSS 17.0 statistical software (SPSS Inc.) was used for all statistical analyses. The χ2 test with Yates correction and Fisher’s exact test were used to examine the relationship between S100A14 or S100A16 expression and clinicopathological variables. The receiver operating characteristics (ROC) curve was constructed to determine diagnostic specificity and sensitivity. The cumulative survival rate was calculated by the Kaplan-Meier method and statistical significance was examined by the log-rank test. Evaluation of the prognostic significance of the clinicopathological factors was performed by univariate and multivariate regression techniques (Cox’s proportional hazards model). Statistical significance was determined as *p* < 0.05.

### Ethical statement

The analysis of human tissues was approved by the Human Research Ethical Committee of Fukushima Medical University (registration number 1203). Written informed consent was obtained from the patients for the publication of this report. This investigation conformed to the principles outlined in the Helsinki Declaration.

## Results

### Evaluation of S100A14 and S100A16 protein expression in breast cancer cells

**Figure 1:**
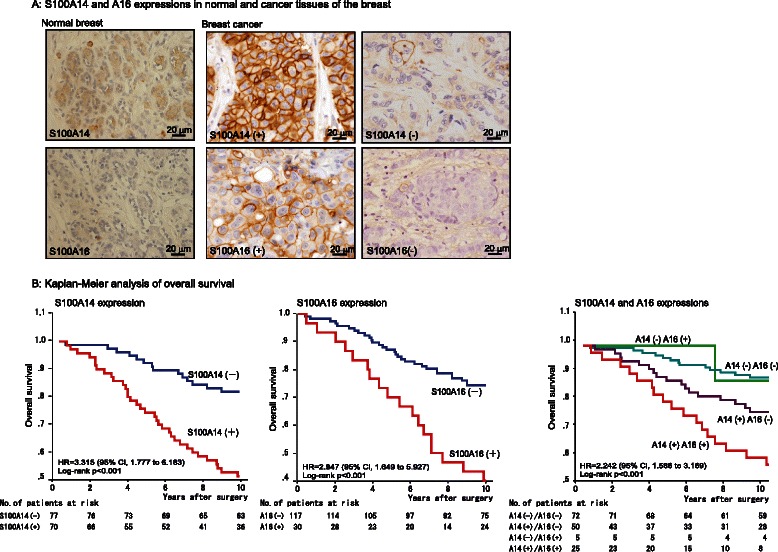
**S100A14 and S100A16 expression in normal and cancerous breast tissues. A**, Representative images of S100A14 and S100A16 protein expression in normal or cancerous breast cancer tissue that was examined using immunohistochemistry. No or faint signals from these proteins were detected in normal epithelial cells of the breast, whereas both proteins were strongly expressed along the cell membrane in about half of breast cancer tissues. **B**, Kaplan–Meier survival analysis of breast cancer patients. Kaplan–Meier survival curves for patients according to the levels of S100A14 and S100A16 expression are shown.

At first, we immunohistochemically screened S100A14 and S100A16 expression in normal and neoplastic cells of each organ (Additional file 3: Figure S3). They were ubiquitously expressed in a variety of expression level and localization, while squamous epithelium strongly expressed these proteins on the cell membrane. To determine the contribution of S100 protein expression to breast cancer, we first used immunohistochemistry to analyze S100A14 and S100A16 protein expression in tissue samples of 167 patients who underwent surgical resection for primary invasive breast cancer. This analysis revealed that S100A14 and S100A16 proteins were expressed mainly on the membrane of breast cancer cells, while no or faint staining was seen in normal epithelial cells of each sample (Figure 1A, representative staining). Immunoreactivity was evaluated using a semiquantitative scoring method that was based on determination of the proportion of positive tumor cells and the staining intensity. With regard to the staining intensity, tumor cells that showed staining of the entire membrane that was as strong as the positive control epidermal squamous epithelium in the same patient, was determined intensity score 2, weaker staining than the control was intensity score 1 and negative staining was considered intensity score 0. The expression levels of S100A14 and S100A16 in tumors were scored by multiplying the percentage of positively stained cells by the intensity score. ROC curves were plotted and analyzed to determine the optimal cut-off values of S100A14 and S100A16 scores. S100A14 and S100A16 showed statistically significant AUCs of 0.660 (*P*= 0.002) and 0.643 (*P* = 0.005). A threshold value of 120 for S100A14 and 55 for S100A16 was the optimal value for maximum sensitivity and specificity, and these values were therefore selected as the cut-off score.

### S100A14 and S100A16 expression and clinicopathological factors of breast cancers

We then correlated the expression of S100A14 and S100A16 proteins with clinicopathological factors (Table 1). Both S100A14 positivity and higher S100A16 expression were significantly associated with younger patient age (<60 years old, *P*= 0.041 and 0.046, respectively), ER-negative status (*P*= 0.014 and 0.030, respectively) and HER2-positive status (*P*= 0.001 and < 0.001. respectively), whereas higher S100A16 expression was also significantly associated with tumor size (*P*= 0.007) and lymph node metastasis (*P* < 0.001). Furthermore, there was a positive correlation between S100A14 expression and S100A16 expression (*P* < 0.001). Co-expression of S100A14 and S100A16 was correlated with younger patient age (<60 years old**,***P*= 0.025), lymph node metastasis (*P*= 0.005), ER-negative status (*P*= 0.008) and HER2-positive status (*P* < 0.001).

**Table 1:** **Association between clinicopathological variables and S100A14 and S100A16**

Clinical data	S100A14			S100A16			S100A14 and S100A16		
	Low expression	High expression		Low expression	High expression		Low expression	High expression	
			*P*			*P*			*P*
	n = 84 (%)	n = 83 (%)		n = 132 (%)	n = 35 (%)		n = 137 (%)	n = 30 (%)	
Patient age (y)									
<60	53(31.7)	65(38.9)	0.047*	88(52.7)	30(18.0)	0.046*	93(55.7)	25(15.0)	0.025*
≥60	31(18.6)	18(10.8)		44(26.3)	5(3.0)		44(26.3)	5(3.0)	
pT									
1	29(17.4)	20(12.0)	0.183	46(27.5)	3(1.8)	0.007^§^	47(28.1)	2(1.2)	0.07
2	49(29.3)	52(31.1)		73(43.7)	28(16.8)		77(46.1)	24(14.4)	
3	6(3.6)	8(4.8)		11(6.6)	3(1.8)		11(6.6)	3(1.8)	
4	0(0)	3(1.8)		2(1.2)	1(0.6)		2(1.2)	1(0.6)	
pN									
0	30(18.0)	22(13.2)	0.153	51(30.5)	1(0.6)	<0.001^§^	51(30.5)	1(0.6)	0.005^§^
1	50(29.9)	50(29.9)		71(42.5)	29(17.4)		76(45.5)	24(14.4)	
2	4(2.4)	10(6.0)		10(6.0)	4(2.4)		10(6.0)	4(2.4)	
3	0(0)	1(0.6)		0(0)	1(0.6)		0(0)	1(0.6)	
TNM staging									
I	25(15.0)	15(9.0)	0.126	37(22.2)	3(1.8)	0.067	37(22.2)	3(1.8)	0.214
II	50(29.9)	50(29.9)		75(44.9)	25(15.0)		80(47.9)	20(12.0)	
III	8(4.8)	14(8.4)		16(9.6)	6(3.6)		16(9.6)	6(3.6)	
IV	1(0.6)	4(2.4)		4(2.4)	1(0.6)		4(2.4)	1(0.6)	
Histological grade									
1	26(15.6)	13(7.8)	0.069	33(19.8)	6(3.6)	0.433	36(21.6)	3(1.8)	0.147
2	36(21.6)	43(25.7)		63(37.7)	16(9.6)		65(38.9)	14(8.4)	
3	22(13.2)	27(16.2)		36(21.6)	13(7.8)		36(21.6)	13(7.8)	
ER									
Negative	23(13.8)	39(23.4)	0.014*	43(25.7)	19(11.4)	0.030*	44(26.3)	18(10.8)	0.008*
Positive	61(36.5)	44(26.3)		89(53.3)	16(9.6)		93(55.7)	12(7.2)	
PgR									
Negative	53(31.9)	50(29.9)	0.826	80(47.9)	23(13.8)	0.721	83(49.7)	20(12.0)	0.618
Positive	31(18.7)	33(19.9)		52(31.1)	12(7.2)		54(32.3)	10(6.0)	
HER2									
score 0 & 1	76(45.4)	56(33.1)	0.001*	114(68.3)	18(10.8)	<0.001*	118(70.7)	14(8.4)	<0.001*
score 2 & 3	8(4.9)	27(16.6)		18(10.8)	17(10.2)		19(11.4)	16(.96)	
S100A16									
low	79(47.3)	53(31.7)	<0.001*						
high	5(3.0)	30(18.0)							

*p <0.05 for χ^2^ test with Yates correction.

^§^*p* <0.05 for Fisher’s exact test.

### Expression of S100A14 and S100A16 predicts the prognosis of breast cancer patients

To analyze the correlation between S100A14 and S100A16 expression with the prognosis of breast cancer patients, Kaplan-Meier survival curves were generated. During the follow-up of 147 patients, 99 patients (67%) survived for 10 years. High positive expression of each protein was correlated with poor outcome (S100A14, *P*= 0.0002; S100A16, *P* = 0.0075, Figure 1B). Double-positive cases that expressed both proteins showed the worst prognosis (*P*= 0.0005). In multivariate analysis, S100A14 expression (*P* < 0.001), PgR (*P*= 0.024) and histological grade (*P* < 0.001) each had significant prognostic value for overall survival (Table 2B). Furthermore, when defined as a single factor, co-expression of S100A14 and S100A16 was determined as an independent prognostic factor (*P*= 0.001) (Table 2C).

**Table 2:** **Univariate and multivariate analysis of overall survival in breast cancer patients**

A. Univariate analysis			
Parameter	*P*	Hazard ratio	95% CI
S100A14 (positive vs negative)	<0.001	3.315	1.777 to 6.813
S100A16 (positive vs negative)	<0.001	2.947	1.640 to 5.927
Co-expression of S100A14 and S100A16 (yes vs no)	<0.001	3.678	2.029 to 6.666
Age (<60 vs ≥ 60 years)	0.475	0.788	0.660 to 2.439
Tumor size (≥2 cm vs < 2 cm)	0.852	1.060	0.576 to 1.951
Lymph node metastasis (yes vs no)	<0.001	2.746	1.770 to 4.261
ER (negative vs positive)	0.003	2.351	1.333 to 4.143
PgR (negative vs positive)	0.040	1.948	1.030 to 3.685
HER2 score (2,3 vs 0,1)	<0.001	2.680	1.492 to 4.815
Histological grade (3 vs 1,2)	<0.001	4.782	2.653 to 8.618
			NS: not significant
**B. Multivariate analysis including each S100A14 and S100A16 expression**			
**Parameter**	***P***	**Hazard ratio**	**95% CI**
S100A14 (positive vs negative)	<0.001	3.397	1.807
S100A16 (positive vs negative)	NS		
Age (<60 vs ≥ 60 years)	NS		
Tumor size (≥2 cm vs < 2 cm)	NS		
Lymph node metastasis (yes vs no)	NS		
ER (negative vs positive)	NS		
PgR (negative vs positive)	0.024	2.107	1.104 to 4.021
HER2 score (2,3 vs 0,1)	NS		
Histological grade (3 vs 1,2)	<0.001	4.316	2.383 to 7.817
			NS: not significant
**C. Multivariate analysis including coexpression of S100A14 and S100A16**			
**Parameter**	***P***	**Hazard ratio**	**95% CI**
Co-expression of S100A14 and S100A16 (yes vs no)	0.001	2.811	1.529 to 5.170
Age (<60 vs ≥ 60 years)	NS		
Tumor size (≥2 cm vs < 2 cm)	NS		
Lymph node metastasis (yes vs no)	NS		
ER (negative vs positive)	NS		
PgR (negative vs positive)	0.049	1.907	1.004 to 3.623
HER2 score (2,3 vs 0,1)	NS		
Histological grade (3 vs 1,2)	<0.001	3.970	2.175 to 7.247
			NS: not significant

### S100A14 and S100A16 expression in human breast cancer cell lines

**Figure 2:**
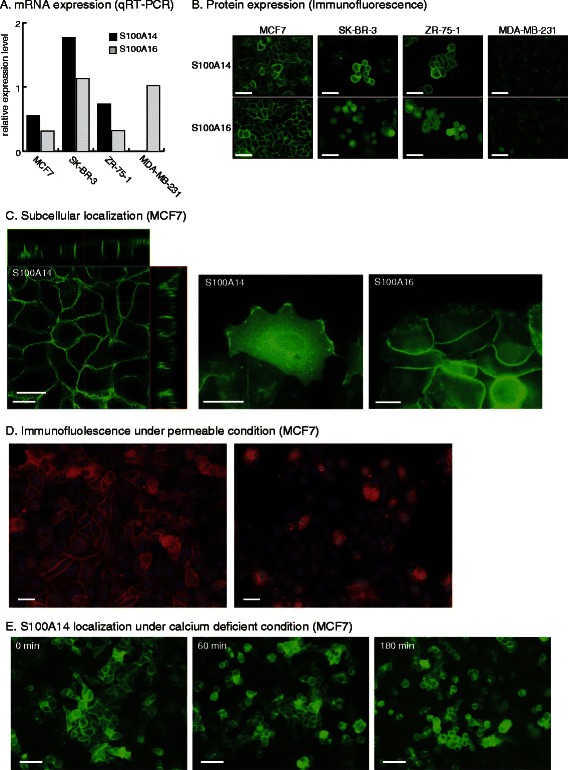
**Expression and localization of S100A14 and S100A16 in human breast cancer cell lines.A**, Relative mRNA expression levels measured by real-time RT-PCR. Expression levels were normalized to β-actin levels within the same sample. **B**, Protein expression of S100A14 and S100A16. Expression levels and subcellular localization were visualized using immunofluorescence staining. Scale bar; 50 μm. **C**, Subcellular localization of the S100A14 protein on the cell membrane. Z-axis images of confluent MCF7 cells were constructed using confocal laser scanning microscopy. Scale bar; 10 μm. **D**, Effect of omission of cell permeabilization on the immunofluorescent staining of S100A14. Following fixation with 4% paraformaldehyde, the cells were treated with or without 0.1% Triton X-100 in PBS prior to staining. Scale bar; 50 μm. **E**, Ca^2^-independent localization of S100A14 on the cell membrane. MCF7 cells transfected with the S100A14-GFP expression vector were observed by fluorescence microscopy over 180 min after addition of 10 mM EGTA in PBS. Scale bar; 50 μm.

To further analyze the role of S100A14 and S100A16 proteins in breast cancer, we next examined the mRNA expression levels of S100A14 and S100A16 in human breast cancer cell lines by using qRT-PCR. MCF7, SK-BR-3 and ZR-75-1 cells expressed both transcripts at high levels, whereas MDA-MB-231 cells expressed only S100A16 mRNA (Figure 2A). Immunofluorescence staining also demonstrated strong expression of S100A14 and S100A16 proteins on the cell membrane of MCF7, SK-BR-3 and ZR-75-1 cells. However, MDA-MB-231 cells did not express either S100A14 or S100A16 proteins (Figure 2B).

### Subcellular localization of S100A14 in MCF7 cells

Since cellular localization of S100 proteins is important for their function, we performed a detailed analysis of the subcellular localization of S100A14 and S100A16 proteins in breast cancer cells. For this study we performed immunofluorescence staining of MCF7 cells, which exhibit distinct polarity. Under an adherent condition, these proteins showed a polarized localization in the cell. Laser scanning confocal microscopy of immunofluorescent stained cells showed that the expression of S100A14 and S100A16 proteins was polarized to the lateral but not to the basal or the apical regions of MCF7 cells (Figure 2C). Time-lapse observation of MCF7 cells transfected with the S100A14-GFP expression vector showed that the protein was static at the cell-cell attachment sites, but it was dynamically moving in waves at the leading edge of the migrating cells (Additional file 4: Video S1). This cell membrane staining using anti-S100A14 and-S100A16 antibodies was mostly undetectable if Triton X-100 pretreatment of the cells, which allows the antibodies to permeate the membrane, was omitted, indicating that these proteins are localized at the cytoplasmic side of the cell membrane (Figure 2D). Time-course observation of MCF7 cells transfected with the S100A14-GFP expression vector under a calcium-deficient culture condition, which was created using 10 mM EGTA in PBS, showed stable localization of S100A14 on the cell membrane for 180 min without loss of cell viability (Figure 2E), indicating that the membrane localization of S100A14-GFP was calcium independent.

### Binding of S100A14 to actin

**Figure 3:**
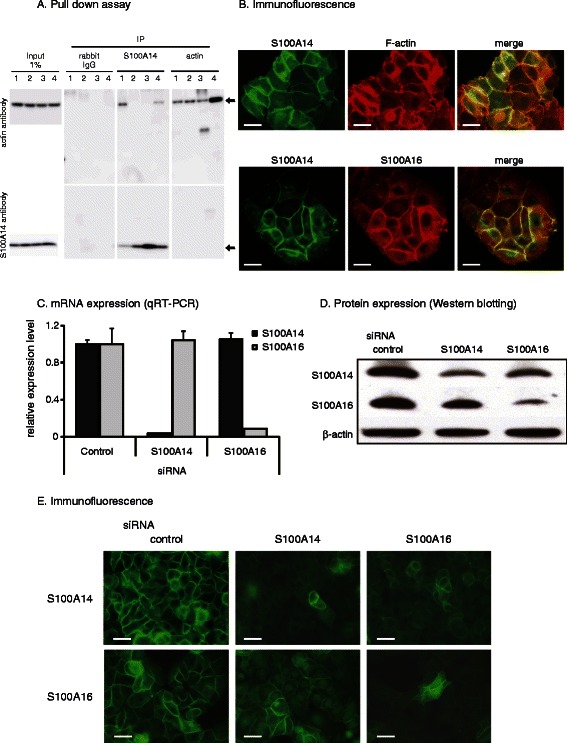
**Interaction of the S100A14 protein with actin and analyses of its functional interaction with S100A16. A**, An extract of MCF7 cells was immunoprecipitated (IP) with control rabbit IgG, anti-S100A14 or anti-actin antibodies. Precipitated and co-precipitated proteins were detected by immunoblotting with anti-S100A14 and anti-actin antibodies. The following were added to the extracts prior to precipitation. CaCl2; 0 mM (lane 1), 0.5 mM (lane 2) or 2 mM (lane 3), or, 2 mM CaCl2 and 10 mM EDTA (lane 4). Arrows indicate actin and S100A14. **B**, Co-localization of S100A14 with polymerized actin and S100A16 in MCF7 cells. Co-immunostaining with the anti-S100A14 antibody and a FITC-labeled second antibody (green), and with rhodamine-conjugated phalloidin (red) showed colocalization of both S100A14 and actin along the cell membrane of MCF7 cells (top). Co-localization of S100A14 and S100A16 was also confirmed in MCF7 cells (bottom). Scale bars; 20 μm. **C**, Analysis of the mRNA expression of S100A14 and S100A16 in MCF7 cells transfected with siRNA for S100A14 or S100A16. Gene knockdown of *S100A14* or *S100A16* does not affect the mRNA expression level of the other S100 protein in MCF7 cells. The relative mRNA expression levels were normalized to the *GAPDH* mRNA expression level. Error bars, + SD, n=3. **D**, Effect of siRNA targeted against S100A14 or S100A16 on the expression of each protein. Western blots of extracts of MCF7 cells transfected with siRNA targeted against S100A14 or S100A16 were probed with antibodies against S100A14, S100A16 and b-actin. E, Expression of S100A14 or S100A16 protein following knockdown of the counterpart molecule. Immunofluorescence analysis of the protein expression and localization of S100A14 and S100A16 in MCF7 cells 48 h after transfection with siRNA targeted against each protein. Scale bars; 10 μm.

To determine if S100A14 binds to actin**,** we performed a pull-down assay of cell extracts of MCF-7 cells using the anti-S100A14 antibody. Binding of S100A14 to actin was detected by Western blotting of the S100A14 immunoprecipitates using specific antibodies (Figure 3A). Though interaction between S100A14 and actin was decreased when 5 mM of EDTA was used along with 2 mM of CaCl2, we coud not detect increased immunoprecipitation of actin with increasing Ca2+ concentrations (0.5 and 2 mM of CaCl2). Reverse immunoprecipitation assay using specific antibody for actin could not detected the interaction between S100A14 and actin. Immunofluorescence analysis demonstrated colocalization of S100A14 and actin in a linear pattern on the membrane at the cell-cell attachment sites (Figure 3B). Immunofluorescence staining of MCF7 cells transfected with the S100A14-GFP expression vector using S100A16 antibody showed colocalization of S100A14 and S100A16 proteins on the cell membrane of MCF7 cells (Figure 3B), although the binding of these proteins to each other was not demonstrated in the pull-down assay (data not shown).

### Functional interaction of S100A14 with S100A16

We examined potential S100A14 and S100A16 Functional interaction by using siRNA-based knockdown methods. Either *S100A14* or *S100A16* targeted siRNA inhibited the mRNA expression of its specific target transcript but did not affect the mRNA expression of the other gene (Figure 3C). At the protein level, Western blot analysis indicated that knockdown of *S100A14* or *S100A16* slightly reduced expression of the other protein, but markedly decreased expression of the target protein (Figure 3D). In contrast, immunofluorescence analysis showed that knockdown of *S100A14* or *S100A16* with their specific siRNAs markedly decreased not only the presence of their target protein on the cell membrane but also the presence of the other protein on the cell membrane (Figure 3E). These experiments suggested that the presence of both proteins is required for the localization of each protein to the cell membrane but that the expression of each protein is independent of the other.

### S100A14 and S100A16 promote cellular invasion

**Figure 4:**
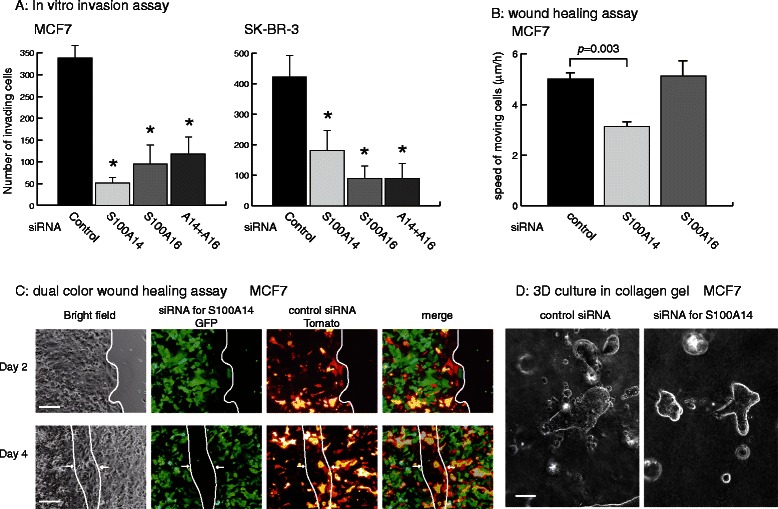
**The effects of S100A14 and S100A16 knockdown on cancer cell invasion and motility. A**, In vitro invasion assay using a Matrigel-coated transmembrane. Invading MCF7 and SK-BR-7 cells were counted 24 h after seeding of knockdown cells (the error bar represent S.D., n=3). **B**, Wound healing assay using MCF7 cells transfected with siRNA for S100A14or S100A16. The wound width was measured, and the distance over which the cells had migrated 48 h after scratching was calculated (the error bars represent S.D., n=3). **C**, A dual-color wound healing assay using MCF7 cells that were stably transfected with GFP or Tomato expression vectors that were further transfected with siRNA for S100A14 or control, respectively. The white lines represent the migration fronts at 48 h after scratching of a mixed culture of cells on a confluent monolayer. Scale bars; 50 μm. **D**, Confocal images of representative spheroids of MCF7 cells grown in a collagen gel for 48 h after transfection of S100A14 or control siRNA. Scale bar; 50 μm.

To analyze the functional roles of S100A14 and S100A16 in cancer cells, we examined the cellular behavior of MCF7 and SK-BR-3 cells transfected with *S100A14* and *S100A16* specific siRNAs. Knockdown of S100A14, S100A16 or of both proteins, all slightly inhibited in vitro cellular proliferation, which was statistically not significant (Additional file 5: Figure S4). Furthermore, knockdown of S100A14 or S100A16 suppressed cellular invasiveness in three assay systems. In the first system, we examined the cellular invasiveness of MCF7 and SK-BR-3 cells by using an in vitro invasion assay with a Matrigel-coated culture insert. Knockdown of S100A14 or S100A16 reduced the number of cells that migrated through 8 μm-sized pores (Figure 4A). The second system that we used consisted of two different wound healing assays that evaluated the effect of S100A14 or S100A16-knockdown on the movement of MCF7 cells. In the standard assay, S100A14 knockdown significantly suppressed cell motility, while motility was not inhibited by S100A16 knockdown (Figure 4B). To confirm the inhibitory activity of S100A14 knockdown on cell movement, we performed a dual-color wound healing assay. This assay is a modified method for monitoring cell movement that uses different-colored MCF7 cells. These cells are stably transfected with either pEGFP (green) or pTomato (red) expression vectors. In a mixed culture of MCF7-GFP cells transfected with S100A14-siRNA and MCF7-Tomato cells transfected with control siRNA, a scratch wound was filled in by cell movements that consisted of red cells in the front line followed by green cells (Figure 4C). Similar results were obtained by using cells with a reverse combination of siRNAs. The third system that we used was an assay of cell invasion using a 3D-culture of tumor spheroids in a collagen gel. After 3 days culture in this system, MCF7 cells that were transfected with control siRNA exhibited features of migrating cells, displaying finger-like projections and satellite cells, while S100A14 knockdown cells did not exhibit any such features of migrating cells (Figure 4D).

## Discussion

This report describes three major findings: 1) S100A14 and S100A16 expression is correlated to poor prognosis in breast cancer patients; 2) The localization of these proteins is limited to the plasma membrane along the lateral surface of the cell, which may be due to their interaction with actin; and 3) S100A14 and S100A16 promote the invasive activity of breast cancer cells. All of these findings indicate that S100A14 and S100A16 expression augments the malignant phenotype of breast cancer.

Our clinicopathological analysis revealed that higher expression levels of S100A14 and S100A16, especially of co-expression of the two proteins, were significantly correlated with factors associated with poor prognosis and with a lower overall survival rate. There have been many reports of S100A14 expression in a variety of cancers [8,10,11,13,20-23], whereas S100A16 has not been well analyzed in clinical cases [24]. In the studies of S100A14 expression, contrasting data regarding its relationship to prognosis, which were based on immunohistochemical methods of S100A14 expression, have been reported; S100A14 expression was correlated with a poorer prognosis in carcinomas of the breast, liver, and stomach [8,9,25] and with a favorable prognosis in colorectal and small intestinal carcinomas [10,11]. One possible reason for these discrepancies is that S100A14 may be able to interact with multiple target proteins such as HER2, MMP2, P53 and receptor for advanced glycation end products (RAGE) [9,12-15] that are involved in various cellular functions, as is the case for other S100 family members. Indeed, our immunohistochemical screening on normal and neoplastic tissues indicates that the molecular functions of S100A14 and S100A16 proteins may differ in different cell types depending on their protein expression levels and their subcellular localization. Therefore, these factors should be taken into consideration when evaluating the expression and potential function of these proteins in cancer cells. In this sense, the immunohistochemical criteria that we used for evaluation of S100A14 and S100A16 positivity in breast cancers, which evaluated the staining pattern and staining intensity of these proteins based on their subcellular localization and potential function in cytoskeletal organization may be a more suitable method for evaluation of these proteins than simply evaluation of total cellular expression of these proteins.

A clinicopathological study of breast cancer was carried out by Xu et al., which immunohistochemically analyzed S100A14 protein expression, and which reported that patient prognosis was poorer in cases in which S100A14 and HER2 were co-expressed compared to cases that did not express either protein. Because of the close correlation between S100A14 and HER2 expression in breast cancer, the prognosis of S100A14-positive patients could be affected by HER2 status when prognosis was analyzed using standard statistical methods. However, our multivariate analysis showed that expression of S100A14, and co-expression of S100A14 and S100A16, are independent prognostic factors for breast cancer. This is the first report in which the clinical significance of S100A14 protein expression as an independent prognostic factor in breast cancer has been demonstrated. In addition, to our knowledge, clinicopathological studies of the expression of S100A16 (except for reports of global expression profiles), or of the co-expression of S100A14 and S100A16 in breast cancer patients have not been reported.

In the present study, the S100A16 protein showed similar expression patterns to S100A14 both in terms of clinical correlations and of cell membrane localization. Our in vitro data indicated that S100A14 and S100A16 are dependent on each other for their membrane localization; when either protein was knocked down, the other protein was not expressed on the membrane although there was no reduction in its mRNA expression level. Therefore, these proteins may require each other for anchorage to the membrane. Indeed, Sapkota et al. demonstrated an interaction between S100A14 and S100A16 proteins in an oral squamous cell carcinoma cell line by using a co-immunoprecipitation method [17]. Thus, interaction between S100A14 and S100A16 proteins may be the reason for the strong correlation of their expression in human breast cancer tissues. The mechanism and function of their interaction, however, are unknown. Thus, S100A14 and S100A16 proteins may promote the malignant nature of breast cancer by acting in cooperation with each other or with other binding proteins on the cell membrane.

The second major findings in this study is the protein localization of S100A14 and S100A16 on the cell membrane, which are commonly found both clinical and experimental studies. Our in vitro study provided a more detailed subcellular localization of these proteins, indicating that they are expressed along the lateral surface of the membrane and at intercellular junctions in the confluent state. These findings suggest that S100A14 and S100A16 might bind to membrane-associated proteins in a similar manner as other S100 family proteins such as S100A10 and A11, which bind to annexins [26-28]. A pull-down assay and subsequent mass spectrometry analysis we previously performed have demonstrated the binding of the S100A14 protein to actin, a major cytoskeletal protein (data not shown). Furthermore, double staining using the S100A14 antibody and phalloidin indicated that S100A14 may bind to membrane-localized polymerized actin, such as cortical actin. Two other molecules; RAGE and HER2, have been identified as binding proteins for S100A14 [9,15]. However, two findings of the present study suggest that S100A14 anchorage to the cell membrane may be mediated by actin assembled on the cell cortex rather than by RAGE or HER2; 1) S100A14 is located at the inner (cytoplasmic) side of the cell membrane, indicating that in these breast cancer cells it cannot be a ligand of a membrane-bound receptor such as RAGE that has a ligand binding region on the outside of the cell and 2) the S100A14 protein is ubiquitously expressed in a wide variety of normal, and neoplastic cells, including squamous epithelial cells, which display no amplification of HER2.

The third important finding of the present study is the invasion-promoting activity of S100A14 and S100A16. Our knockdown studies demonstrate that these molecules can promote cell motility and invasive activity of breast cancer cells. There have been some reports regarding the contribution of S100A14 functions to the malignant nature of cancer cells [8,9,13,15]. Although the molecular mechanisms are not fully understood, S100A14 can promote cancer cell invasion by regulating MMP2 transcription in a p53-dependent manner and it can also induce cell proliferation and apoptosis by binding to RAGE, resulting in stimulation of RAGE-dependent signaling cascades. Our findings indicate a novel molecular mechanism of S100A14 that may promote cancer invasion by binding to actin to activate cell movement. In addition, as shown in the dual-color wound healing assay, most of the moving cells at the migration front of a migrating population of cells were S100A14 positive cells. In contrast, at the rear of this front, cancer cells including S100A14-positive and-negative cells appear to follow the migrating leader cells by maintaining cell-cell attachments, whether they express S100A14 protein or not. These cellular and molecular dynamics suggest that the movement of neoplastic epithelial cells requires not only binding of S100A14 to cytoskeletal actin but also additional conditions at the migrating front.

## Conclusion

Our findings suggest that S100A14 and S100A16 protein expression may be predictive biomarkers for poorer prognosis of breast cancer patients. Because S100A14 and S100A16 can promote cancer cell motility and invasion via modulation of cytoskeletal dynamics, these proteins could be novel target molecules for therapeutic strategies in breast cancer patients.

## Additional files

Additional file 1: Figure S1. Absorption test of S100A14 and S100A16 antibodies mixed with their corresponding antigenic recombinant proteins. A, Immunohistochemistry using paraffin sections of squamous epithelium of skin and of breast cancer. B, Western blotting using an extract of MCF7 cells.
Additional file 2: Figure S2. Comparison of the gene knockdown efficacy among siRNAs with three different sequences for each mRNA of S100A14 and S100A16. After 48 h from transfection with siRNA, MCF7 cells on a culture side was stained according to the immunofluorescence method. Scale bars; 20 μm (A) and 40 μm (B). The sequence of siRNA were S100A14 No. 1: 5′-CCUUCUGAGCUACGGGACCUGGUCA-3′, S100A14 No. 2: 5′-GAGUUCAGGAGUUUCUGGGAGCUGA-3′, S100A14 No. 3: 5′-CCAACGCAGAGGAUGCUCAGGAAUU-3′, S100A16 No. 1: 5′-CCAAUCAUGAUGGGCGCAUCAGCUU-3′, S100A16 No. 2: 5′-AAGGCAGUCAUUGUCCUGGUGGAAA-3′ and S100A16 No. 3: 5′-CAGGGAACCGGAAGGCUGCGGAUAA-3′.
Additional file 3: Figure S3. Immunohistochemical analysis of S100A14 protein expression in normal and cancerous tissues. The figures show representative images of S100A14 protein expression. The subcellular localization of this protein is different in normal tissues; it is in the membrane in the epidermis, in both the membrane and the cytoplasm in the colon and is in the cytoplasm in renal tubules (Upper figures). In cancers, the expression and the localization of the S100A14 protein are different depending on the tissue of origin (Lower figures). SqCC: squamous cell carcinoma, RCC: renal cell carcinoma. The table shows the number of various cancer cases with strong expression of the S100A14 protein as assessed by using this immunohistochemical method. Scale bars; 500 μm.
Additional file 4: Video S1. Time-lapse video microscopy of MCF7 cells stably transfected with GFP-tagged S100A14.
Additional file 5: Figure S4. The effects of S100A14 and S100A16 knockdown on in vitro cell growth of MCF7 and SK-BR-3 cells. Growth of the cells was determined by a cell viability (XTT) assay after treatment with siRNAs targeted towards S100A14, S100A16, or both proteins for 48 h (error bars represent S.D., n=3).

## Abbreviations

MMP: Matrix metalloproteinase
RAGE: Receptor for advanced glycation end products
PBS: Phosphate buffer saline
DMEM: Dulbecco’s modified Eagle medium
GFP: Green fluorescent protein
RNAi: RNA interference
siRNA: Small interfering RNA
FBS: Fetal bovine serum
qRT^-^PCR: quantitative real-time PCR
CHAPS: 3-(3-cholamidepropyl) dimethylammonio-1-propanesulphonate
EDTA: Ethylenediaminetetraacetic acid
HEPES: 4-(2-hydroxyethyl)-1-piperazineethanesulfonic acid
DMP: dimethyl pimelimidate dihydrochloride
FITC: Fuorescein isothiocyanate
DAPI: 4’,6-diamidino-2-phenylindole
SDS-PAGE: Sodium lauryl sulfate-polyacrylamide gel electrophoresis
ER: Estrogen receptor
PgR: Progesterone receptor
3D: 3 dimensional
