# Supplementary figures and images for: Co-expression of S100A14 and S100A16 correlates with a poor prognosis in human breast cancer and promotes cancer cell invasion
Source: BMC Cancer. 2015 Feb 13;15:53. doi: 10.1186/s12885-015-1059-6 (PMC4348405; doi:10.1186/s12885-015-1059-6)

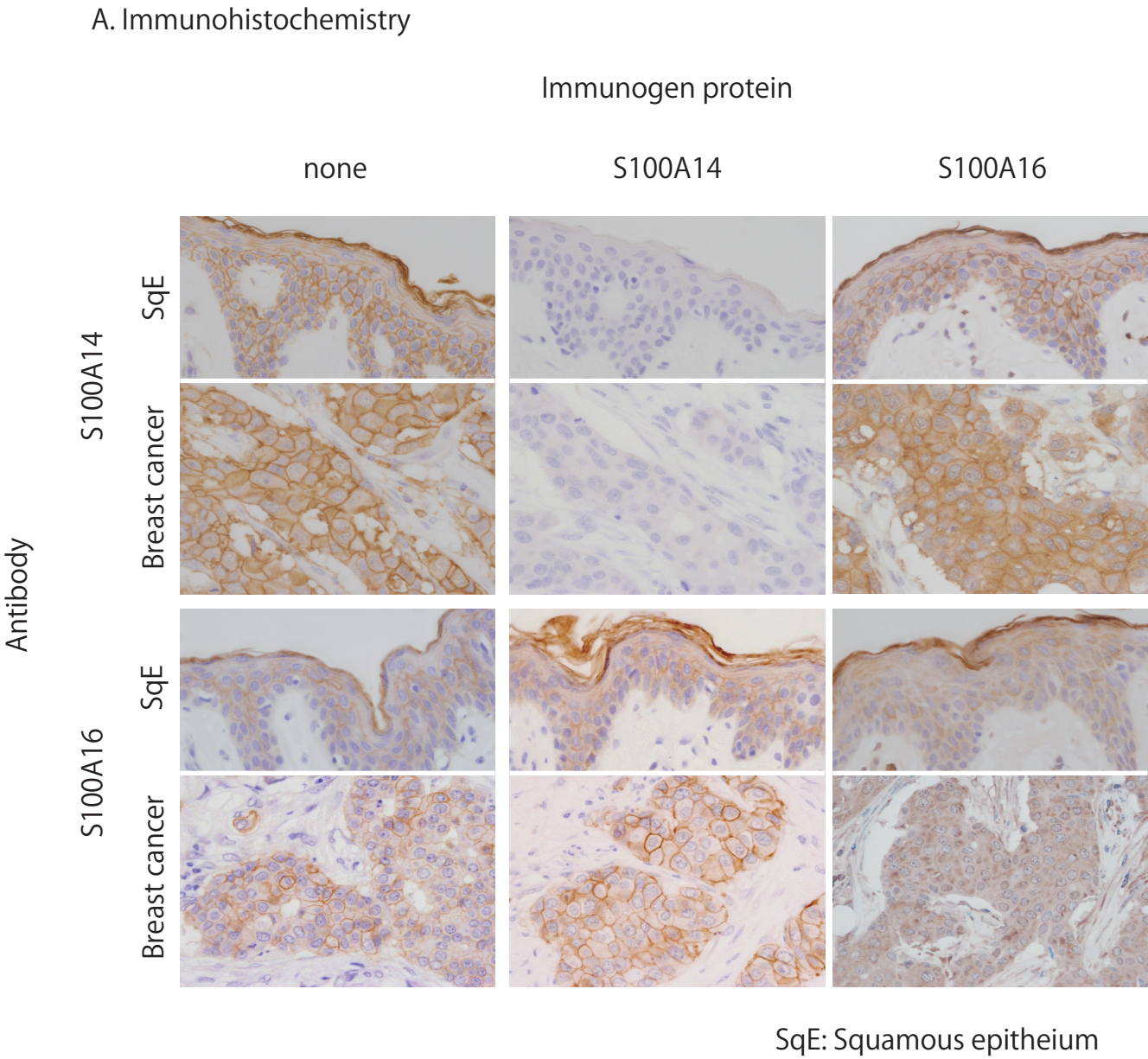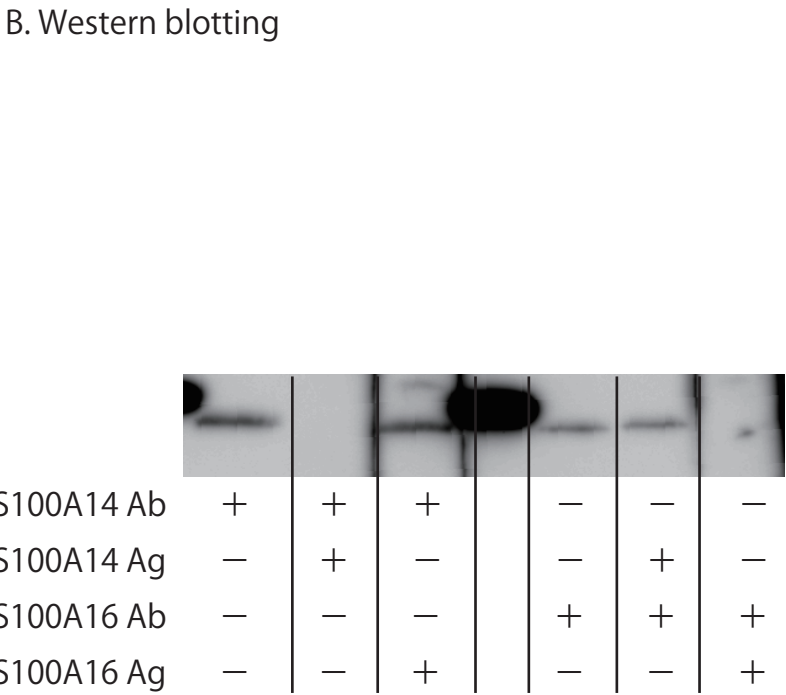

Supplement: Additional file 1: Figure S1. — Absorption test of S100A14 and S100A16 antibodies mixed with their corresponding antigenic recombinant proteins. A, Immunohistochemistry using paraffin sections of squamous epithelium of skin and of breast cancer. B, Western blotting using an extract of MCF7 cells. [file 12885_2015_1059_MOESM1_ESM.pdf]

A. siRNA for S100A14

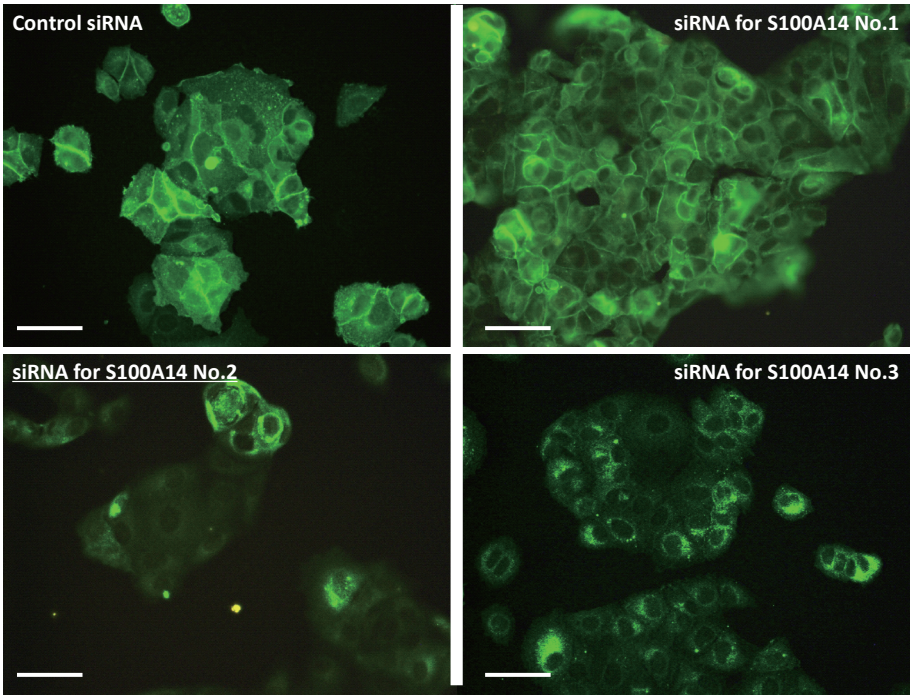

B. siRNA for S100A16

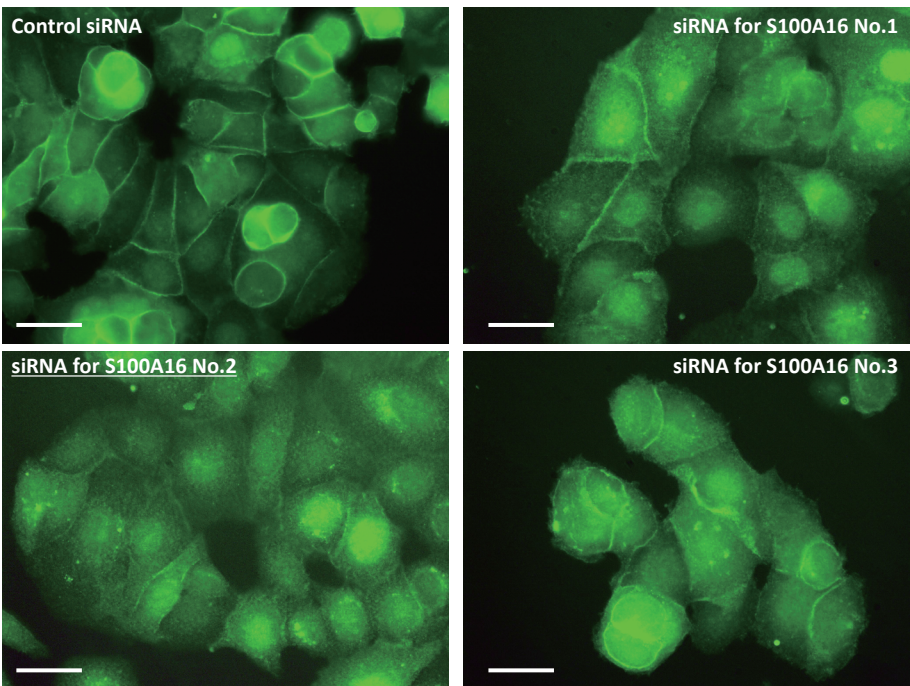

Supplement: Additional file 2: Figure S2. — Comparison of the gene knockdown efficacy among siRNAs with three different sequences for each mRNA of S100A14 and S100A16. After 48 h from transfection with siRNA, MCF7 cells on a culture side was stained according to the immunofluorescence method. Scale bars; 20 μm (A) and 40 μm (B). The sequence of siRNA were S100A14 No. 1: 5′-CCUUCUGAGCUACGGGACCUGGUCA-3′, S100A14 No. 2: 5′-GAGUUCAGGAGUUUCUGGGAGCUGA-3′, S100A14 No. 3: 5′-CCAACGCAGAGGAUGCUCAGGAAUU-3′, S100A16 No. 1: 5′-CCAAUCAUGAUGGGCGCAUCAGCUU-3′, S100A16 No. 2: 5′-AAGGCAGUCAUUGUCCUGGUGGAAA-3′ and S100A16 No. 3: 5′-CAGGGAACCGGAAGGCUGCGGAUAA-3′. [file 12885_2015_1059_MOESM2_ESM.pdf]

in vitro growth (XTT assay)

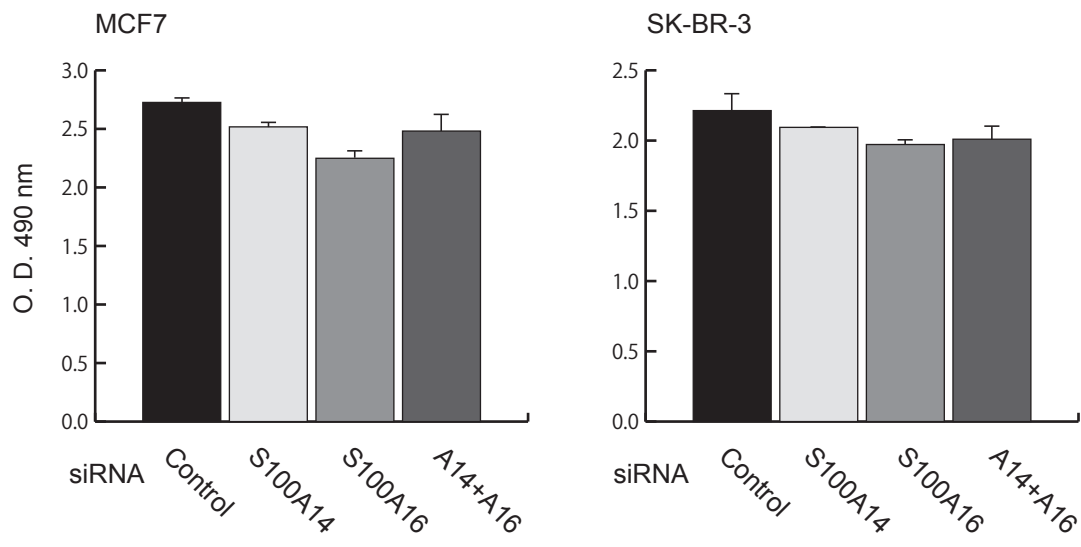

Supplement: Additional file 3: Figure S3. — Immunohistochemical analysis of S100A14 protein expression in normal and cancerous tissues. The figures show representative images of S100A14 protein expression. The subcellular localization of this protein is different in normal tissues; it is in the membrane in the epidermis, in both the membrane and the cytoplasm in the colon and is in the cytoplasm in renal tubules (Upper figures). In cancers, the expression and the localization of the S100A14 protein are different depending on the tissue of origin (Lower figures). SqCC: squamous cell carcinoma, RCC: renal cell carcinoma. The table shows the number of various cancer cases with strong expression of the S100A14 protein as assessed by using this immunohistochemical method. Scale bars; 500 μm. [file 12885_2015_1059_MOESM3_ESM.pdf]
